# CHALLENGES OF VERY LATE LIFE: PHYSICAL AND MENTAL HEALTH, CAREGIVING, AND AGING PERCEPTIONS

**DOI:** 10.1093/geroni/igad104.0916

**Published:** 2023-12-21

**Authors:** Daniela Jopp, Kathrin Boerner

## Abstract

The very old are the fastest growing population in developed countries, yet research on this age group remains limited. There are, however, specific challenges of very old age that differ from those in earlier late life phases. Very old individuals and their families may thus have unique needs and may require adapted services. In this symposium, we address selected challenges associated with reaching very old age, including physical and mental health issues, as well as implications of both receiving and providing care. Ribeiro and colleagues report on centenarians’ health and morbidity profiles using the Portuguese census, by comparing individuals aged 100 years old in 2011 and 2021. Jopp and colleagues present findings from the first national Swiss centenarian study on centenarians’ health, cognition, and well-being. Gomes da Rocha and colleagues investigate depressive symptoms in Swiss centenarians during the COVID-19 pandemic, showing notably elevated scores. Kim and colleagues focus on aging perceptions and their correlates among dyads of very old parents and their old children from South Korea, highlighting the impact of the care experience for both dyad members. Gallagher and colleagues examine how old children of very old parents with dementia manage behavioral and psychological symptoms through specific strategies, offering insights on how to better support family care givers of very old dementia patients. In sum, this symposium offers novel insights on specific challenges encountered in very old age, by the individual and their families, and proposes potential pathways towards better understanding and handling of such challenges.

